# The cognitive effects of adjunctive repetitive transcranial magnetic stimulation for late-onset depression: a randomized controlled trial with 4 week follow-up

**DOI:** 10.3389/fpsyt.2023.1240261

**Published:** 2023-08-08

**Authors:** Wei-gang Pan, Xiao-yue Hu, Dan-di Zhu, Li Li, Feng Bao, Li Ren, Pei-xian Mao, Xin Ma, Yan-ping Ren, Yi-lang Tang

**Affiliations:** ^1^Beijing Key Laboratory of Mental Disorders, National Clinical Research Center for Mental Disorders & National Center for Mental Disorders, Beijing Anding Hospital, Capital Medical University, Beijing, China; ^2^Advanced Innovation Center for Human Brain Protection, Capital Medical University, Beijing, China; ^3^Department of Psychiatry, Xicheng District Pingan Hospital, Beijing, China; ^4^Department of Psychiatry and Behavioral Sciences, Emory University School of Medicine, Atlanta, GE, United States; ^5^Mental Health Service Line, Atlanta VA Medical Center, Decatur, GE, United States

**Keywords:** late-onset depression, transcranial magnetic stimulation, cognition, random controlled trial, cognitive impaiment

## Abstract

**Objectives:**

Cognitive impairment is common and linked to poor outcomes in patients with late-onset depression (LOD). The cognitive effects of repetitive transcranial magnetic stimulation (rTMS) for LOD are not well understood. This study aimed to investigate the effects of rTMS on cognitive function in elderly patients with LOD.

**Methods:**

In total, 58 elderly patients (aged 60 to 75 years) with depression were enrolled and randomly assigned to an active rTMS group or a sham group. The participants received active or sham rTMS over the left dorsolateral prefrontal cortex for 4 weeks, 5 days a week, at a frequency of 10 Hz rTMS and 120% of the motor threshold (MT). Cognitive function was assessed using the Repeatable Battery for the Assessment of Neuropsychological Status (RBANS) at baseline, the end of the 4 week treatment period, and at the 4 week follow-up.

**Results:**

The active rTMS group showed significant improvements in immediate memory and attention scores on the RBANS compared to the sham group. However, no significant differences were observed between the two groups in other cognitive domains assessed by the RBANS. No serious adverse events related to rTMS treatment were observed.

**Conclusion:**

Treatment with 120% MT rTMS was associated with improvement in cognitive defects related to the active phase of LOD. These findings suggest that rTMS could provide early improvements in cognitive function in clinical settings for elderly patients with LOD.

**Clinical trial registration**: https://www.chictr.org.cn/showproj.html?proj=40698, identifier ChiCTR1900024445.

## 1. Introduction

Late-onset depression (LOD) is a significant global concern (1, 2), and cognitive impairment is a prominent issue associated with LOD, potentially representing a core pathophysiology rather than a secondary symptom of depression (3). Notably, cognitive dysfunction and emotional state dysregulations often coexist independently in LOD (4–6). It is believed that cognitive impairment influences the effectiveness of antidepressant treatment (7). Previous studies have shown that cognitive function can predict the outcomes of antidepressant treatment in patients with LOD and that lower cognitive function is associated with poorer response to antidepressant treatment (8). Cognitive impairment in geriatric depression is characterized by poor response to antidepressants and functional disability (5). A meta-analysis also suggested that cognitive impairment is associated with unfavorable prognosis (9). This is consistent with the observation of several studies that cognitive impairments predict poor response to antidepressant treatment in elderly individuals with depression (8, 10, 11). Conversely, active enhancement of cognitive function could improve the efficacy of antidepressant therapy (6, 10). However, there is a scarcity of clinical trials investigating the treatment of cognitive impairment in patients with LOD.

Addressing cognitive impairment in patients with LOD is crucial to improve their outcomes. However, despite extensive research on new pharmacological treatments over the last two decades, no strong evidence has emerged for improving cognitive impairments in patients with LOD (6, 12). Antidepressants remain the mainstay of treatment for LOD, but approximately 30% of LOD patients do not respond adequately to the available first-line antidepressants (13). Treatment resistance is estimated to affect about 50% of depressed patients receiving appropriate antidepressant therapy, and over 10% of these patients remain resistant to various psychopharmacological interventions. In addition, there is a significant risk of relapse (up to 85% of cases) or chronicization (about 20% of cases) (14). Moreover, most antidepressants do not effectively improve cognitive functions such as information processing, verbal memory, decision-making speed, reaction inhibition, and attentiveness, as shown by a large study involving 1,008 patients with major depressive disorder (15). In fact, according to a systematic review, antidepressants were associated with a two-fold increased risk of cognitive decline (16). Therefore, there is a need for the development and evaluation of nonpharmacological treatments. Cognitive remediation, which aims to enhance cognitive skills and functioning through structured exercises, may be a promising alternative or adjunctive treatment for patients with LOD (10, 17). However, further studies are required to confirm its efficacy and long-term benefits.

Repetitive transcranial magnetic stimulation (rTMS) has been found to be effective and well-tolerated for treating depression in younger adults (14, 18). Compared to pharmacological therapy, rTMS is noninvasive, well tolerated, and generally safe (1). However, the efficacy of rTMS for LOD patients is not well-established, although some studies have suggested its efficacy and tolerability (19, 20). Previous studies have shown that older age is linked to a poorer response to rTMS (21) and there is a relative lack of research on rTMS in older patients (13). Possible reasons for the reduced response to rTMS in patients with LOD include brain atrophy and suboptimal rTMS parameters (21). Therefore, it is important to adjust the rTMS protocol according to the specific characteristics of patients with LOD, such as cortical thickness and resting motor threshold (22). Recent studies have shown that increasing the intensity and frequency of rTMS stimulation may improve the outcomes in patients with LOD and achieve response rates comparable to those observed in younger patients (23). The FDA protocol for rTMS involves using 10 Hz at 120% of the motor threshold (MT) intensity for 20–30 sessions over 4–6 weeks (1). However, most of the published randomized controlled trials on rTMS for LOD did not strictly follow the protocol and used lower intensities or shorter durations (1, 20, 22). Therefore, more research is needed to determine the optional rTMS parameters for patients with LOD that follow the FDA guidelines and take into account individual differences, including the use of stimulation intensities above 110% of the resting MT, which is a metric used to individualize treatment parameters (13).

Although the efficacy of rTMS for alleviating depressive symptoms is well documented, its cognitive effects in patients with LOD are not well understood and require more research (24). An early study has shown that rTMS is associated with improved cognitive performance and spontaneous brain activity in healthy individuals (25). Another study showed that rTMS is not associated with any negative impact on neuropsychological functioning within current therapeutic parameters (14). However, the evidence regarding the cognitive benefits of rTMS has also been inconsistent across specific cognitive tests (26). This is supported by a recent meta-analysis of randomized controlled trials with sham designs in individuals with neuropsychiatric illnesses (including depression, bipolar disorder, and schizophrenia), which did not find clear benefits of rTMS on various cognitive domains, such as global cognitive function, verbal and visual memory, attention, executive function, processing speed, and visuospatial ability, compared to sham rTMS (27). In contrast, some evidence suggests that rTMS may enhance cognitive performance in individuals with treatment-resistant depression (28, 29), especially in domains such as scanning vision, mental motor speed, and spatial orientation abilities (30). The above findings suggest that rTMS may have more selective cognitive effects depending on the specific clinical context and cognitive task.

Over the past decade, in clinical trials focusing on rTMS for depression, the left dorsolateral prefrontal cortex (DLPFC) has been the target of excitatory stimulation (14). A decrease in DLPFC activity has been linked to depression and cognitive impairment, and rTMS indirectly induces electrical currents in this region (31, 32). By stimulating the DLPFC, rTMS increases the excitability of cortical neurons, induces long-term potentiation, and enhances the plasticity of the brain (33). It is possible that DLPFC could alter the cognitive control network (CCN), a regulatory system that modulates the function of psychological and cognitive systems (32). Studies have shown that rTMS can exert antidepressant effects by regulating CCN function and influencing cognitive control in emotion regulation (32, 34). The use of rTMS to target the DLPFC is an established and well-tolerated treatment for depression (14, 35).

Studies have shown the beneficial effects of rTMS on the mood of geriatric patients, but the evidence for its effects on the cognitive function of these patients is limited (14). To the best of our knowledge, no previous study has examined the relationship between rTMS and cognitive performance in patients with first-episode drug-naïve LOD. We hypothesized that compared to the sham rTMS group, the active rTMS group would exhibit significant improvements in cognitive function. To test this hypothesis, we conducted a randomized, double-blind, sham-controlled trial. In this study, the active rTMS group received 20 sessions of rTMS at 120% of the MT intensity, targeting the left DLPFC. The sham group received sham stimulation that mimicked the sensation of rTMS but did not induce actual cortical stimulation. Cognitive functioning was assessed using the Repeatable Battery for the Assessment of Neuropsychological Status (RBANS), which measures various cognitive domains, including immediate memory, delayed memory, visuospatial, attention, and language in comparison to the sham rTMS group.

## 2. Materials and Methods

### 2.1. Participants

A total of 58 patients with LOD were recruited between June 2019 and March 2020 from Beijing Anding Hospital, affiliated with Capital Medical University in Beijing, China. LOD was defined as the onset of depressive symptoms at 60 years of age or older. The criteria for inclusion were: (i) age 60 years or older; (ii) diagnosis of depressive episodes according to the ICD-10 diagnostic criteria; (iii) Hamilton Depression Rating Scale-17 score of 17 or higher. Patients with (i) any other major mental disorders other than depression; (ii) a history of severe brain trauma, seizures, or other neurological disorders; (iii) intracranial implant; (iv) major medical or neurological illness that could potentially confound the study results; and (v) A Mini Mental Status Exam (MMSE) score over 20 for those with a primary education level or a score over 24 for those with middle school education level or above were excluded (36, 37).

This study was approved by the Ethics Committee of Beijing Anding Hospital affiliated with Capital Medical University [number: (2019) Scientific Research No. (40)]. Informed consent was obtained from all participants before enrollment. The trial was registered with the Chinese Clinical Trial Registry (ChiCTR1900024445).

### 2.2. Design

This was a randomized, double-blind, and sham-controlled trial. Participants were randomly assigned to either the active rTMS or the sham rTMS group in a 1:1 ratio using a random number table. The randomly generated code was sealed in an envelope and kept by the neuromodulation center to maintain the blinding of treatment conditions for both study investigators and clinicians. The duration of the trial was 8 weeks, which included 4 weeks of 20 daily rTMS sessions (excluding weekends) and a 4 week follow-up period without rTMS sessions.

The sample size of the study was set at 60 based on power analysis and an assumed type I error rate of 0.05. The estimated remission rates for the active rTMS group and the sham rTMS group were 39.4 and 6.9%, respectively, based on previous studies using rTMS (38). The allocation of participants between the treatment groups was set at a 1:1 ratio.

### 2.3. Interventions

An rTMS system with an eight-coil device (Magstim, United Kingdom) was used for the analysis. The rTMS treatment targeted the DLPFC using the “5 cm rule.” According to this rule, the DLPFC was situated 5 cm anterior (in a parasagittal line) to the motor cortex.

In the active rTMS group, the following standardized dose was administered: stimulation frequency of 10 Hz, intensity set at 120% of the MT, a pulse train duration of 4 s, an inter-train interval of 56 s, and a total stimulation time of 20 min per day. Each session consisted of 800 TMS pulses delivered to the DLPFC using the eight-coil device. In the sham rTMS group, the internationally recognized pseudo-stimulation method was used to keep the horizontal coil upright (39). The coil used in the sham stimulation had the same appearance, stimulation frequency, stimulation time, and stimulation period as the real stimulation coil; however, the angle of the coil ensured that the magnetic field did not pass through the skull. Instead, an electrical current was generated, resulting in an ineffective stimulus.

The doctor altered the dose of the prescribed drugs (escitalopram or sertraline) according to the patient’s clinical condition during the 8 week course of antidepressants. Short-term benzodiazepines may be prescribed for patients who experience severe sleep disturbances, anxiety, or agitation.

### 2.4. Assessments

The severity of depressive symptoms was assessed using the 17-item Hamilton Depression Rating Scale (HDRS) at baseline, as well as after 4 and 8 weeks of the treatment period.

Cognitive function was evaluated using the RBANS. The RBANS is a commonly used cognitive assessment tool in clinical trials that measures various cognitive domains in elderly patients with depression (8, 20). It assesses five cognitive domains: attention (measured through digital span and coding), language (evaluated through picture naming and semantic fluency), delayed memory (assessed using list recall, list recognition, story recall, and figure recall), immediate memory (measured by list learning and story memory), and visuospatial ability (evaluated through figure copy and line orientation). The total score is calculated by summing the five index scores, and the cutoff point for the total score is typically set at 90 to 109, with lower scores indicating poorer cognitive function. The RBANS assessment was conducted from 24 to 48 h after the completion of rTMS sessions. The RBANS is a relatively easy-to-administer test that typically takes about 20 min to complete.

### 2.5. Statistical analysis

Data were analyzed using SPSS 25 software (IBM, Armonk, NY, United States). Means and standard deviations (SD) were calculated for the data. Demographic variables were analyzed using independent sample *t*-tests for continuous variables and chi-square tests for categorical variables. The final analysis was conducted based on the intent-to-treat (ITT) principle. The dependent variable was the cognitive domain measured by the RBANS test. Repeated-measures ANOVA was used to examine the effects of group (active vs. sham) as the between-participants component and the time of the RBANS test (baseline, 4 week treatment, 4 week follow-up) serving as the within-participants factor on cognitive functions. Contrast tests were conducted to further explore the interaction effects between group and time. Statistical significance was determined using a two-tailed significance level set at *p* < 0.05.

## 3. Results

### 3.1. Demographic information

**Figure 1:**
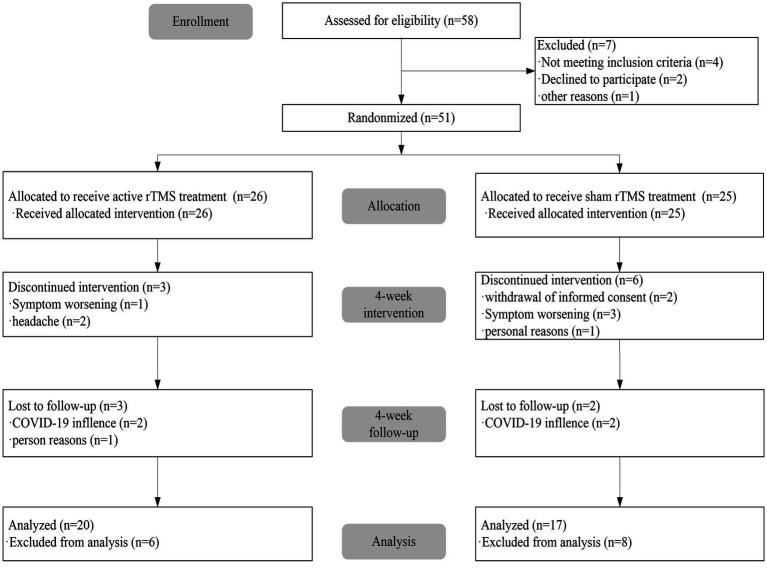
The CONSORT graphic shows how participants moved through the trail.

A total of 58 participants were enrolled in the study, 7 of whom did not proceed to randomization owing to ineligibility. The flowchart of participant enrollment and allocation is presented in Figure 1.

Participants in the ITT sample were randomized to the rTMS group with 26 active participants and the sham rTMS group with 25 participants. The baseline demographic characteristics of patients with LOD are summarized in Table 1. A total of 51 (87.9%) participants completed the acute course of treatment. No significant differences were observed in age, education level, gender, and baseline HDRS score between the two treatment groups. In terms of medication, the proportions of patients on sertraline and escitalopram were comparable between the two groups at the end of the 4 week treatment. There was no significant difference in the dosage of sertraline and escitalopram between the groups (Table 2).

**Table 1:** Demographic characteristics of 51 patients with late-onset depression.

	Active rTMS (*n* = 26)	Sham rTMS (*n* = 25)	*t*/*χ*^2^	*P*
Age (years)	66.2 ± 4.0	66.1 ± 4.3	0.048	0.962
Education (years)	7.4 ± 2.7	7.2 ± 2.5	0.565	0.141
Gender: Male/Female	11/15	9/16	0.213	0.645
Baseline HDRS score	31.2 ± 1.3	30.1 ± 1.4	0.315	0.578
Antidepressant medications				
Escitalopram	16	8	3.204	0.073
Sertraline	7	11		

rTMS, repetitive transcranial magnetic stimulation.

**Table 2:** The dosage of antidepressants between active and sham rTMS group.

Antidepressants	Baseline	4 week treatment	4 week follow-up
Sertraline			
Active rTMS	40.91 ± 12.61	177.27 ± 23.60	184.09 ± 20.21
Sham rTMS	46.43 ± 9.45	182.14 ± 18.90	196.43 ± 9.45
*t*/*z*	−0.990	−0.459	−1.746
*P*	0.337	0.653	0.100
Escitalopram			
Active rTMS	9.69 ± 0.85	19.69 ± 1.25	19.69 ± 1.25
Sham rTMS	8.75 ± 1.89	20.00 ± 0.00	20.00 ± 0.00
*t*/*z*	1.694	−0.669	−0.669
*P*	0.104	0.492	0.492

rTMS, repetitive transcranial magnetic stimulation.

### 3.2. Cognitive function

**Figure 2:**
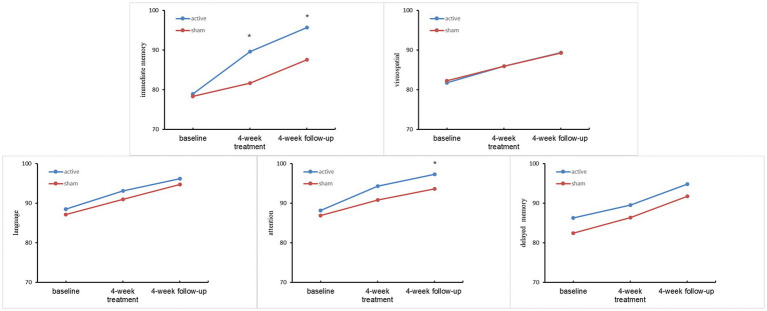
Comparison of performances on cognitive tasks between the two groups.

The changes in cognitive function are shown in Figure 2. Repeated-measures ANOVA was used to test the differences in cognitive function after the 4 week treatment and the 4 week follow-up period. Statistically significant differences were observed in immediate memory at the end of the 4 week treatment (*F* = 4.629, *p* = 0.038) and 4 week follow-up (*F* = 4.684, *p* = 0.037) period. In terms of attention, a significant difference was found between the active and sham rTMS groups after the 4 week follow-up period (*F* = 7.273, *p* = 0.011). However, no significant improvements were observed in visuospatial function, language function, or delayed memory after the 4 week treatment or the 4 week follow-up period (all *p* > 0.05; Table 3).

**Table 3:** Comparison of cognitive performance between two patients receiving rTMS and sham treatment.

	Baseline	4 week treatment	4 week follow-up
RBANS-immediate memory			
Active rTMS	78.95 ± 2.78	89.65 ± 2.52	95.70 ± 2.54
Sham rTMS	78.29 ± 3.02	81.65 ± 2.74	87.59 ± 2.76
*F*	0.026	4.629	4.684
*P*	0.874	0.038	0.037
RBANS-visuospatial			
Active rTMS	81.75 ± 2.29	85.95 ± 2.23	89.35 ± 2.21
Sham rTMS	82.24 ± 2.49	85.94 ± 2.41	89.24 ± 2.39
*F*	0.021	0	0.001
*P*	0.887	0.998	0.972
RBANS-language			
Active rTMS	88.55 ± 1.69	93.15 ± 1.53	96.20 ± 1.23
Sham rTMS	87.10 ± 1.78	91.01 ± 1.62	94.73 ± 1.29
*F*	0.35	0.928	0.716
*P*	0.558	0.342	0.403
RBANS-attention			
Active rTMS	88.15 ± 1.41	94.30 ± 1.24	97.30 ± 0.92
Sham rTMS	86.88 ± 1.53	90.82 ± 1.34	93.65 ± 1.00
*F*	0.37	3.637	7.273
*P*	0.574	0.065	0.011
RBANS-delayed memory			
Active rTMS	86.30 ± 1.75	89.50 ± 1.69	94.80 ± 1.46
Sham rTMS	82.47 ± 1.89	86.35 ± 1.84	91.77 ± 1.58
*F*	2.214	1.59	1.994
*P*	0.146	0.216	0.167

rTMS, repetitive transcranial magnetic stimulation; RBANS, repeatable battery for the assessment of neuropsychological status.

### 3.3. Safety

During the trial, no serious side effects were observed in the active or sham rTMS groups. However, some side effects were reported by patients receiving rTMS. Specifically, in the active rTMS group, seven and three participants reported experiencing headache and nausea, respectively, whereas in the sham rTMS group, four participants each reported experiencing headache and nausea.

## 4. Discussion

To the best of our knowledge, this is the first double-blind, randomized, sham-controlled trial to explore the effects of adjunctive rTMS treatment on cognition in patients with LOD. We found that rTMS treatment targeting the left DLPFC at 120% of MT improved several cognitive domains in patients with LOD. Both the active and sham rTMS groups experienced similar adverse effects. The results showed that immediate memory and attention significantly improved in patients with LOD after rTMS, suggesting that rTMS may improve cognitive functioning in patients with LOD without significant adverse effects.

Studies have shown that LOD is associated with more prominent cognitive impairment compared to that observed in younger patients with depression (7). Some studies have suggested that cognitive symptoms during acute depression may affect the effectiveness of antidepressant treatment (40, 41). Memory and attention dysfunction are the main cognitive symptoms of geriatric depression, which often impair social functioning outcomes (7, 9, 42). A recent meta-analysis suggested that attention deficits in geriatric depression are associated with poor prognosis (9). Our finding showed that immediate memory improvement is especially pertinent to rTMS treatment for LOD. This is consistent with a previous study wherein the rTMS was related to an improvement in immediate memory in treatment-resistant depression beyond practice effects (43). Another study demonstrated that young adults with depression showed an improvement in immediate memory following rTMS treatment (44). However, it should be noted that we did not observe significant improvements in attention after 4 weeks of treatment, but we did find significant improvements at the 4 week follow-up, suggesting that rTMS may have a delayed effect on attention in patients with LOD. This is supported by previous studies that showed that rTMS improves selective attention without affecting mood (45). Similarly, another study found improved attention in patients with major depressive disorder in just 3 months of follow-up, indicating delayed neuropsychological effects of rTMS (46). Studies suggest that the improvement in attention may occur later compared to other cognitive functions (47). However, the exact mechanism underlying the effects of rTMS on improving attention is unclear.

We did not observe improvements in visuospatial function, language function, and memory in patients receiving rTMS treatment, which is in line with previous reports (48, 49). This suggests that the therapeutic effects of rTMS on depressive symptoms may not be directly linked to changes in these specific cognitive domains. The relationship between cognitive impairment and depressive symptoms in patients with LOD is complex, and it is challenging to establish a clear causal relationship between the two (7, 42, 50). The current data does not allow us to infer the underlying mechanism of how rTMS enhances cognitive function (28). It is possible that rTMS alleviates depression and subsequently enhances cognitive performance, or vice versa. Alternatively, rTMS may have independent effects on both cognitive function and depressive symptoms by modulating various neural pathways and brain areas involved in these processes (43).

The mechanism underlying the cognitive effects of rTMS is unclear, and multiple theories have been proposed. Neurobiochemical changes, alterations in neuroconnectivity, and modulation of neuroinflammatory processes may all play a role in the cognitive improvements observed with rTMS (51). Animal studies have shown that rTMS promote neurogenesis, regulate the transmission pathway of brain-derived neurotrophic factor, and increase the expression of N-methyl-D-aspartate (52, 53). These chemical changes might affect the intrinsic and external properties of neurons, alter neurotransmitter and receptor expression, and boost the firing of neurotrophins. Such changes can profoundly affect synaptic plasticity, including long-term potentiation, which is associated with learning and memory (53). The enhancements in cognitive function observed with rTMS may be related to alterations in the neurochemistry and blood flow of the DLPFC and its functionally connected regions (32). For example, rTMS applied to the DLPFC may stimulate and optimize the function of the precuneus, a part of the frontoparietal network, resulting in increased functional connectivity between the precuneus and the default mode network (28). This may explain the relationship between pre-treatment cognitive measurements and the effectiveness of rTMS for depressive disorders. We hypothesize that this potential neural pathway may account for the enhanced attention after rTMS treatment. However, further investigation is needed to determine the specific mechanisms underlying the effects of rTMS on cognitive function in patients with LOD.

This study has several limitations that should be taken into account. First, the use of rTMS as an adjunctive treatment made it difficult to isolate the specific effects of rTMS on cognition, as the potential influence of antidepressants could not be ruled out. Second, the sample size was relatively small to carry out subgroup analysis or generalize the findings to a larger population. Third, the limited number of assessment points (baseline, 4 weeks, and 8 weeks) and the short duration of the study (4 + 4 weeks) rendered it impossible to evaluate the potential longer-term cognitive effects of rTMS. Future studies should explore the effectiveness of rTMS treatment in a larger sample over a longer period of time by using rTMS as the sole treatment for LOD.

## 5. Conclusion

In conclusion, this study demonstrated that adjunctive rTMS treatment with 120% of MT can improve the cognitive function of patients with LOD. These findings suggest that rTMS could be a valuable addition to the treatment options for LOD in clinical practice. However, more research is needed to confirm our findings on the cognitive effects of rTMS in this patient population using rTMS as a monotherapy. Future studies should aim to include larger sample sizes and longer treatment durations and explore the specificity of cognitive improvements attributed to rTMS.

## Data availability statement

The original contributions presented in the study are included in the article/supplementary material, further inquiries can be directed to the corresponding authors.

## Ethics statement

The studies involving humans were approved by the Ethics Committee of Beijing Anding Hospital affiliated with Capital Medical University [number: (2019) Scientific Research No. (40)]. Informed consent was obtained from all participants before enrollment. The studies were conducted in accordance with the local legislation and institutional requirements. The participants provided their written informed consent to participate in this study.

## Author contributions

W-gP and X-yH: investigation and writing-original draft. D-dZ, FB, and LR: collected study data. LL and X-yH: data analysis. Y-pR and XM: study design, supervision, and manuscript revision. P-xM: funding acquisition, project administration, writing-review, and editing. XM: study design and revised the manuscript. Y-lT: data interpretation and critical revisions of the manuscript. All authors contributed to the article and approved the submitted version.

## Funding

This study was supported by Beijing Municipal Science & Technology Commission (No. Z191100006619105).

## Conflict of interest

The authors declare that the research was conducted in the absence of any commercial or financial relationships that could be construed as a potential conflict of interest.

## Publisher’s note

All claims expressed in this article are solely those of the authors and do not necessarily represent those of their affiliated organizations, or those of the publisher, the editors and the reviewers. Any product that may be evaluated in this article, or claim that may be made by its manufacturer, is not guaranteed or endorsed by the publisher.
